# A Simplified Print-in-Place Redesign of the Open-Source Federica Prosthetic Hand

**DOI:** 10.3390/bioengineering13090998

**Published:** 2026-08-27

**Authors:** Levi Tynan, Osura Perera, Thomas Purss, Benjamin Brandwood, Daniele Esposito, Upul Gunawardana, Ranjith Liyanapathirana, Gaetano Gargiulo

**Affiliations:** 1 School of Engineering, Design and Built Environment, Western Sydney University, Penrith, NSW 2751, Australia; l.tynan@westernsydney.edu.au (L.T.); o.perera@westernsydney.edu.au (O.P.); 22032814@student.westernsydney.edu.au (T.P.); b.brandwood@westernsydney.edu.au (B.B.); u.gunawardana@westernsydney.edu.au (U.G.); r.liyanapathirana@westernsydney.edu.au (R.L.); 2 The MARCS Institute for Brain, Behaviour and Development, Western Sydney University, Penrith, NSW 2751, Australia; 3 Department of Information and Electrical Engineering and Applied Mathematics, University of Salerno, Via Giovanni Paolo II, 132, I-84084 Fisciano, Italy; daesposito@unisa.it; 4 Translational Health Research Institute, Western Sydney University, Penrith, NSW 2751, Australia; 5 The Ingham Institute for Applied Medical Research, Liverpool, NSW 2170, Australia

**Keywords:** prosthetic hand, 3D printing, print-in-place design, grip force measurement

## Abstract

Utilising the open-source design of the Federica Prosthetic Hand, we introduce a redesigned print-in-place version that minimises assembly requirements without sacrificing functionality. The original Federica hand has a complicated, time-consuming assembly process. The print-in-place design removes the process of assembling the hand, making it easier for new users to use the design. The approach results in a 21% reduction in the bill of materials and a 21% reduction in weight. Though the 8 N force of the original Federica hand was not reached in this trial, the measured results show that under the same test conditions, the print-in-place hand can match the performance of the original. In a single-specimen analysis, the print-in-place hand produced a higher peak force in four of eight orientations. The redesigned prosthetic hand includes functional joints fully integrated into its structured design with a locking mechanism, eliminating the need for several metal bolts, and a revised dorsal finger contour, reducing the requirement for additional support structures during printing. As with the original hand, the print-in-place prosthetic is open source for anyone to access.

## 1. Introduction

Upper limb prosthetic devices restore function and independence to amputees. However, the high cost, complexity, and assembly requirements of most current prosthetic hands are major barriers to widespread adoption [1,2,3], especially in resource-constrained environments. The increased availability and affordability of benchtop 3D printers have held the promise of developing low-cost, customisable prosthetic devices at the fingertips of individuals and healthcare professionals, who can now fabricate the devices at the local level with minimal setup requirements [4,5,6].

Independent characterisation has established the limits of the printed prosthetics. Llop-Harillo et al. benchmarked four widely used affordable tendon-driven hands—IMMA, Limbitless, Dextrus and InMoov—using participants operating each device through a common adaptor. They found the best-performing design reached 57% of the functionality of the human hand, with contact friction and thumb opposition identified as the principal limitations [7]. Reported grip forces for this class of device consequently fall well below those of commercial powered prostheses, and this gap is a known characteristic of affordable tendon-driven designs rather than a property of any individual hand [8]. Work on sensor integration has sought to recover some of the functional shortfall by other means, for example through low-cost piezoresistive sensing of contact and grip force [9].

Among these 3D-printed prosthetic hands, the Federica hand [10] stands out as an open-source, low-cost model intended for ease of fabrication and accessibility to the masses [10,11,12,13,14,15,16]. Despite its advantages, the original Federica hand model still relied on assembling multiple individual printed components, requiring several screws and fasteners, resulting in longer fabrication times and a higher potential for assembly errors.

To overcome these issues, this work focuses on redesigning the Federica hand into a print-in-place (PIP) design, whereby both the mechanism and joints are incorporated directly into the 3D-printed model. Such a strategy eliminates post-print assembly, reducing manufacturing complexity. The tendons are still manually assembled to the printed hand to accommodate the actuation system. Print-in-place designs have been used in prosthetics before [17], though taking an existing prosthetic design with many joints and converting it into a PIP design is quite novel.

### Federica Hand

**Figure 1:**
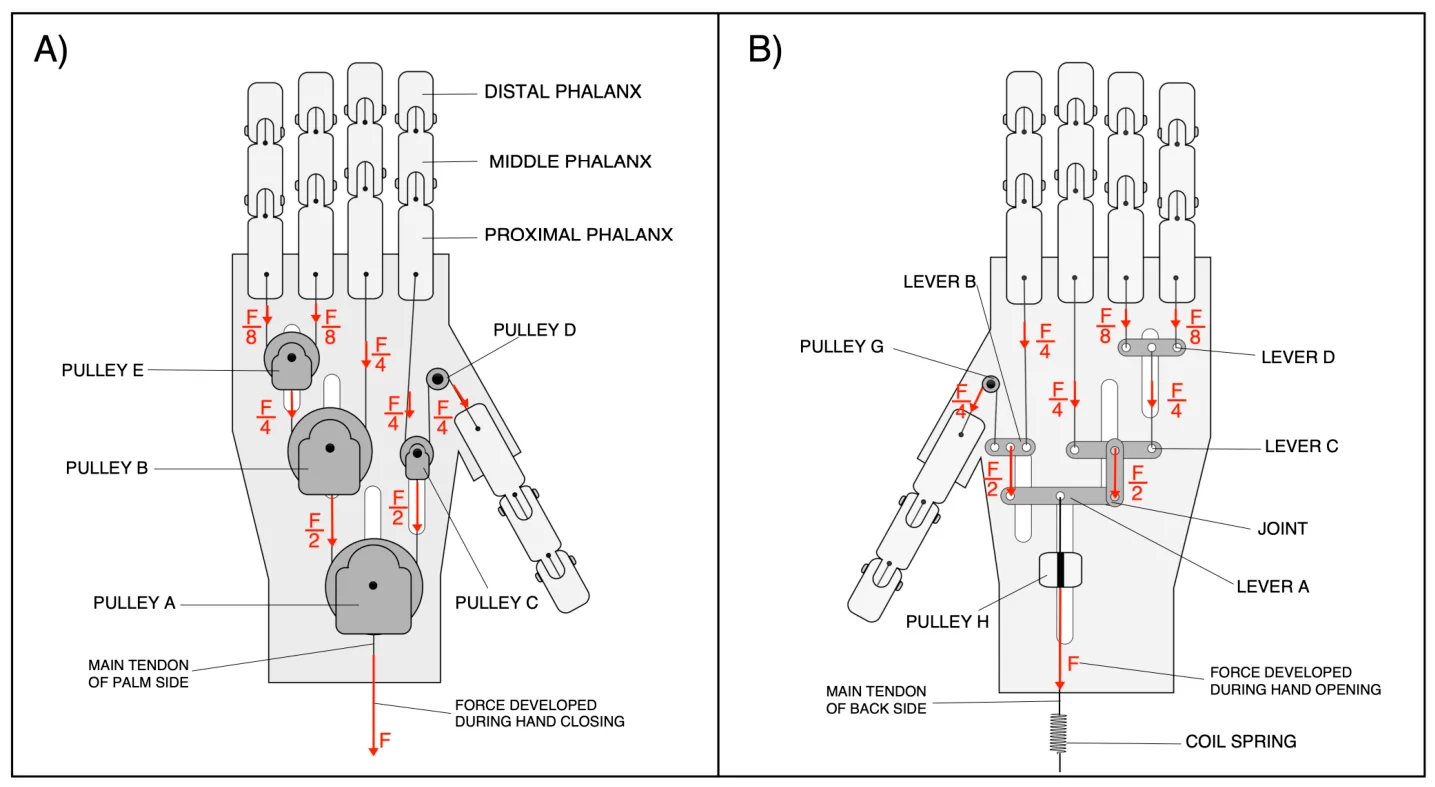
The “Federica” hand illustration: (**A**) Palmar side view of the flexion differential mechanical system, with the forces transferred through the different pulley states shown by the red text and the direction indicated by the red arrows; (**B**) dorsal view of the extension differential of the mechanical system, with the forces transferred through the different lever states shown by the red text and the direction indicated by the red arrows, adapted from [10].

The Federica hand is an open-source, 3D-printed prosthetic device that is designed to provide affordable mobility solutions for individuals with upper limb amputations [10]. It consists of a 3D-printed PLA (polylactic acid) hand assembly and a single servomotor. 3D models of the hand are available as open-source STL files, allowing for easy modifications. The Federica hand consists of five fingers, each equipped with three phalanges, resulting in a total of 15 degrees of freedom. A single servomotor with approximately 2.94 Nm (30 kg/cm) torque effectively controls all 15 phalanges via inelastic cables and pulleys with various ratios, as indicated in Figure 1. The hand is controlled using force-myographic (FMG) signals, which detect muscle contraction using piezoresistive sensors [16]. Proximal phalanges of all fingers (except the thumb) are connected to the palm using a single 80 mm-long M4 screw inserted through the ‘knuckles’, and the thumb is connected to the palm using a 15 mm-long M4 screw. All the other joints of the fingers are connected using 15 mm-long M4 screws. The pulleys are connected using 20 mm-long M3 screws. A 1 mm diameter Nylon rope is used as the ‘tendon’ of the hand. The mechanical arrangement includes a pair of primary tendons: one on the palmar side to close the hand (flexing the fingers) and another on the dorsal side to open the hand (extending the fingers). A coil spring with a low elastic constant is integrated into the back of the hand to enable full finger flexion, thereby enhancing grip strength [14].

The Federica hand demonstrates the ability to perform various daily tasks, including grasping differently shaped objects and lifting loads of up to 1 kg with a rapid activation time of around half a second, triggered by FMG muscle sensors. Powered by a 7.4 V, 3000 mAh battery, it can operate autonomously for an entire day during typical daily activities. As originally proposed and tested, the hand’s mean grip force is 8.80 N, with the middle phalanges exerting 2.65 N and the distal phalanges exerting 1.66 N on average. The force-transfer ratio of the mechanical system is approximately 12.85%, and the average energy dissipation for a complete closing–opening cycle is 106.80 Nmm, which is lower than that of many commercial prostheses [10].

## 2. Materials and Methods

### 2.1. “Print-in-Place” Federica Hand

**Figure 2:**
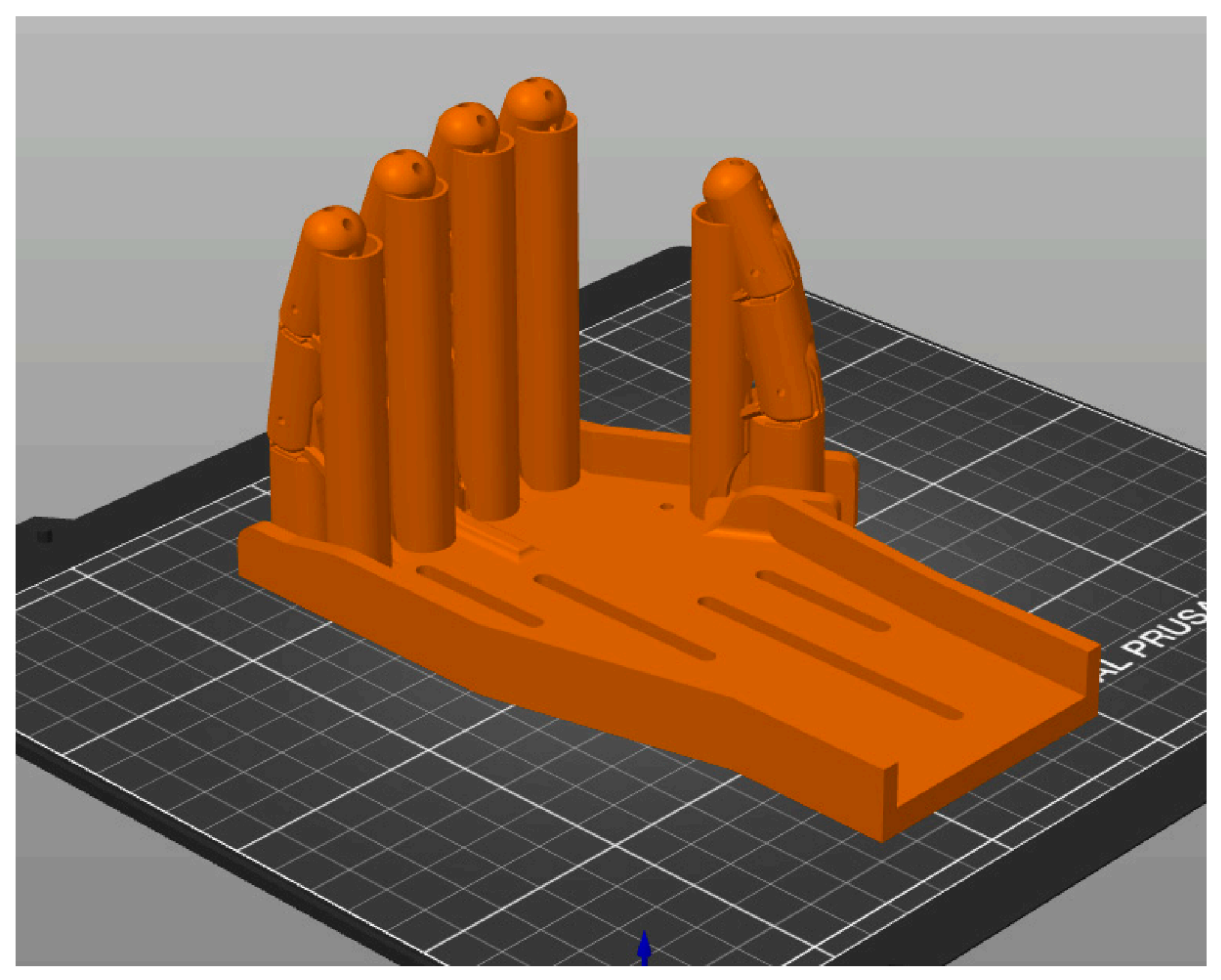
PIP Federica hand with built-in column supports.

The Federica hand was originally constructed by printing components separately and assembling them with screws of varying lengths and appropriate nuts. The assembly process of the hand is both time-consuming and labour-intensive. Additionally, printing many disconnected pieces of similar shapes and sizes (i.e., the fingers’ individual parts) creates confusion during assembly and requires detailed instructions and part labelling. To eliminate these limitations, the design of the Federica hand was changed to a print-in-place (PIP) model, as shown in Figure 2, placed directly on the 3D-printer bed. Print-in-place is a 3D printing method utilised to print assemblies with functionally mobile joints in a single unit, printed in one session. The PIP Federica hand was developed using the original Federica hand available in STL format, modelled and updated using the SolidWorks software, version R2025x FD03. The PIP Federica hand is also available in the Supplementary Materials below for anyone to access and print with the original Federica hand.

**Figure 3:**
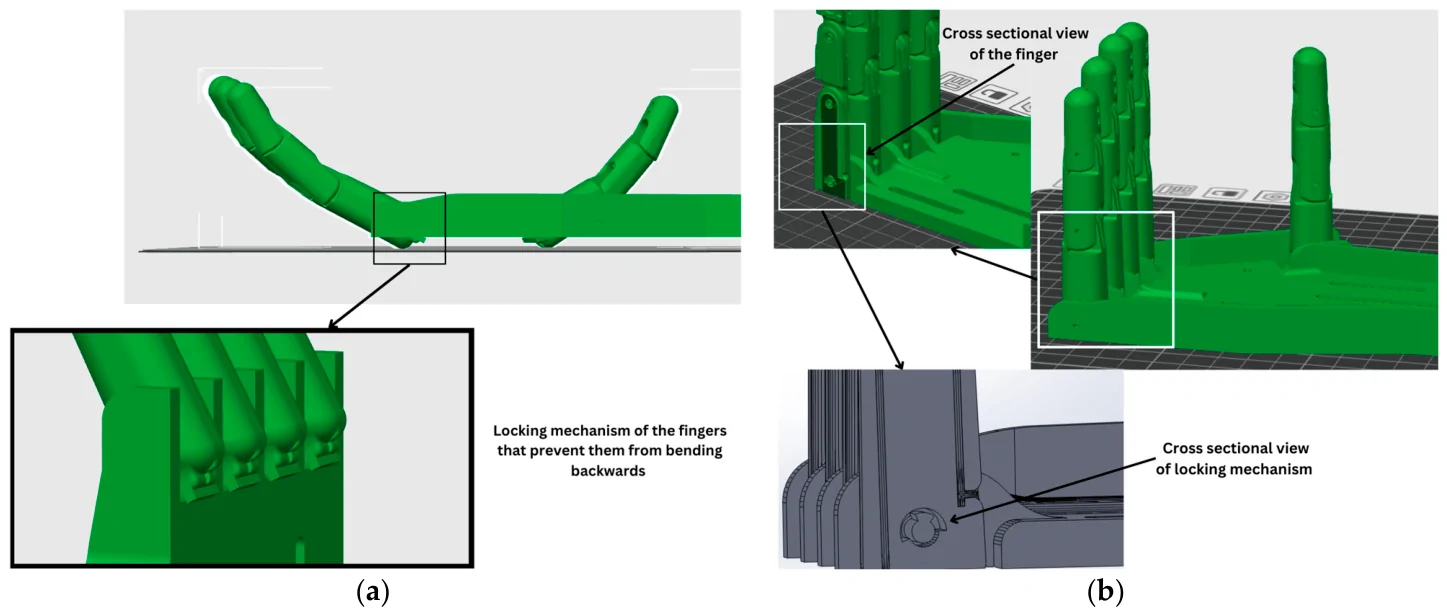
(**a**) The locking mechanism of the fingers in the original Federica hand that prevents them from bending backwards. (**b**) New locking mechanism for the PIP Federica hand.

The initial design changes concentrated on using minimal support to print the hand. As shown in Figure 2, the dorsal side of the palm of the PIP Federica hand sits flat on the printing bed. This was achieved by removing the finger lock mechanism (shown in Figure 3a, which, in the previous design, extended below the palm).

The new locking mechanism is designed so that the fingers can be placed vertically while printing; this is shown in Figure 3b. The new mechanism is inside the base of the finger itself, incorporated into the joint between fingers and the palm.

**Figure 4:**
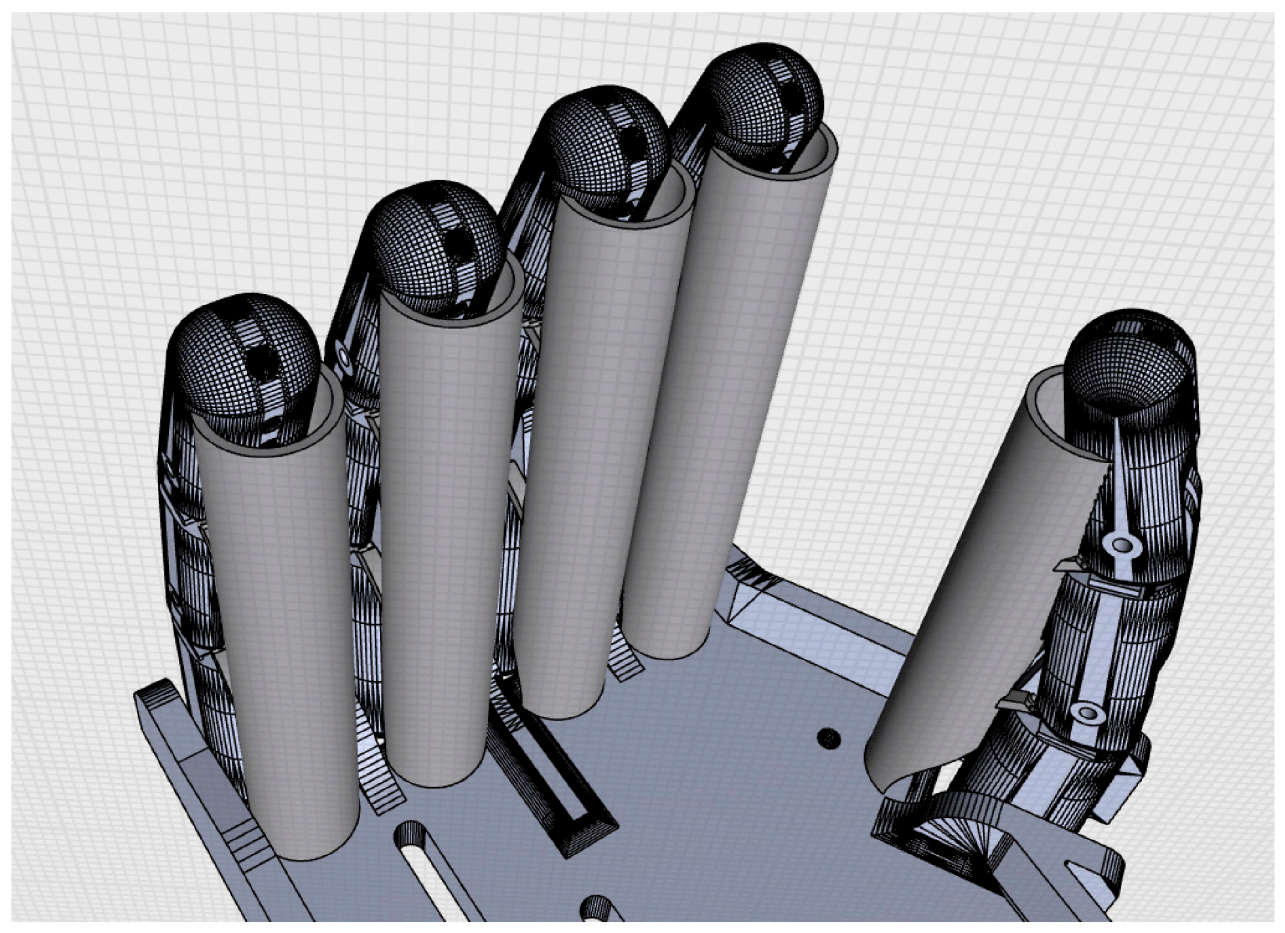
3D model of the Federica hand with the new locking mechanism and column supports.

Both the original design and the PIP design were printed and tested using PLA to ensure consistency across the test. All design versions are printed with a 0.2 mm layer height and a 15% infill density. The support columns (shown in solid grey in Figure 4) can be removed by applying pressure with pliers on either side of the top of the columns. Small supports are extended from the support column in Figure 4 to help keep the joints suspended along the phalanges while printing.

**Figure 5:**
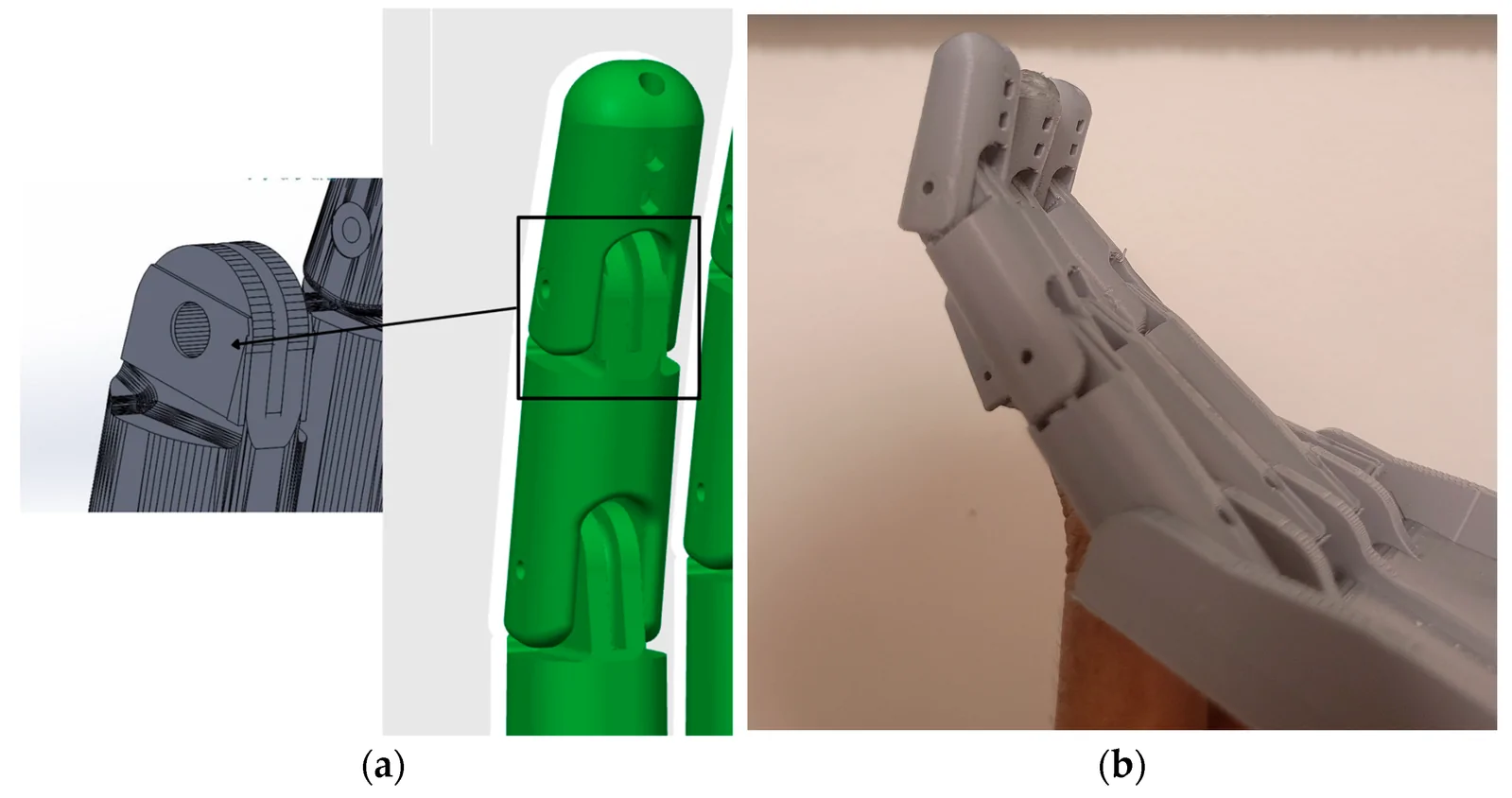
(**a**) Close-up of the reduced clearance and press-fit connection between the middle and distal phalanges. (**b**) Final print of the PIP Federica hand with the press-fit joint of the middle and distal phalanges.

Lastly, to ensure the correct rotation of the distal phalange joints whilst gripping objects, there is a reduction in the clearance distance in the joint that connects the middle and distal phalanges and an added boss included in the edge of the middle phalange, as shown in Figure 5. This part has a thickness of 0.2 mm, which creates a press fit between the middle and distal phalanges. The joints can now be bent easily, but still need some pressure to work against the press-fit joint. This way, the hand can make a smooth gripping motion. The grip force of the Federica hand with the new locking mechanism and reduced clearance of the middle and distal phalanges is compared with a reconstruction of the original Federica hand.

The final design was made available on the Open Science Framework as an STL file, with or without supports.

**Figure 6:**
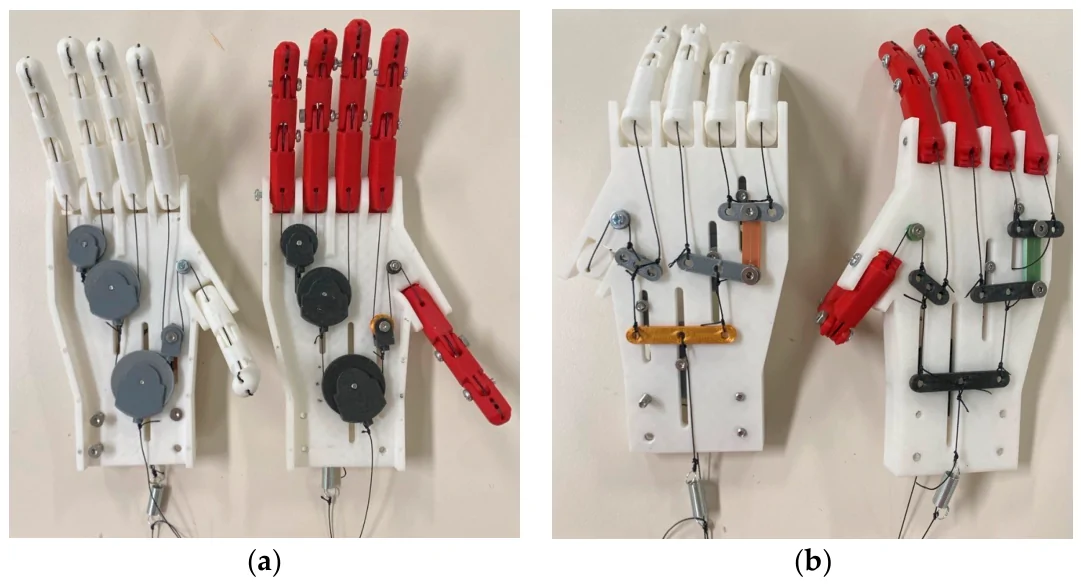
(**a**) Palmar view of the original Federica hand and PIP Federica hand side-by-side. (**b**) Dorsal view of the original Federica hand and PIP Federica hand side-by-side.

A side-by-side comparison of the original Federica hand and the PIP Federica hand can be seen in Figure 6. Both hands are fully assembled with the pulleys and tension spring in place.

### 2.2. Manufacturing Analysis

The PIP file was printed on the Bambu Lab A1, Bambu Lab, Shenzhen, China and took 5 h to print, including the pulley and levers. The print was run 10 times with zero failures, and all support was removed without damaging the print. Table 1 compares key fabrication processes for both the PIP Federica hand and the original Federica hand. Bambu Studio v 2.8.2.61 was used to generate the files for the analysis. Both Federica hands use the same pulleys and levels. The pulleys also need fasteners, which is why the PIP still needs 5 fasteners. The fasteners required for the original Federica hand include ten 15 mm M4 bolts, one 20 mm M4 bolt and one 80 mm M4 bolt, totalling twelve fasteners for the hand assembly alone. For the pulleys, five 20 mm M3 bolts were used.

Table 2 compares the support removal time of the PIP Federica hand to the fastener assembly time for the original Federica hand. This does not include the assembly time for the pulleys and levers, which we timed to take 58 min and 19 s. So, including the support removal time, the Federica hand has a total assembly time of 59 min and 29 s. For the original hand, that makes a total assembly time of 1 h 10 min and 34 s.

### 2.3. Bill of Materials Comparison

The bill of materials costs for both hands are presented in Table 3 and Table 4, as of 23 August 2026. Two totals were provided, one for purchasing all items for initial fabrication and the other for the unit cost, assuming you have stock on hand. The cost of the original Federica was 10.47 AUD, compared to 8.29 AUD for the PIP Federica, a cost reduction of 21%. If you had to buy all the items at full price, the cost of the original Federica would be 62.41 AUD, compared to 51.08 AUD for the PIP Federica, providing an 18% reduction.

### 2.4. Federica Hand Servomotor Setup

**Figure 7:**
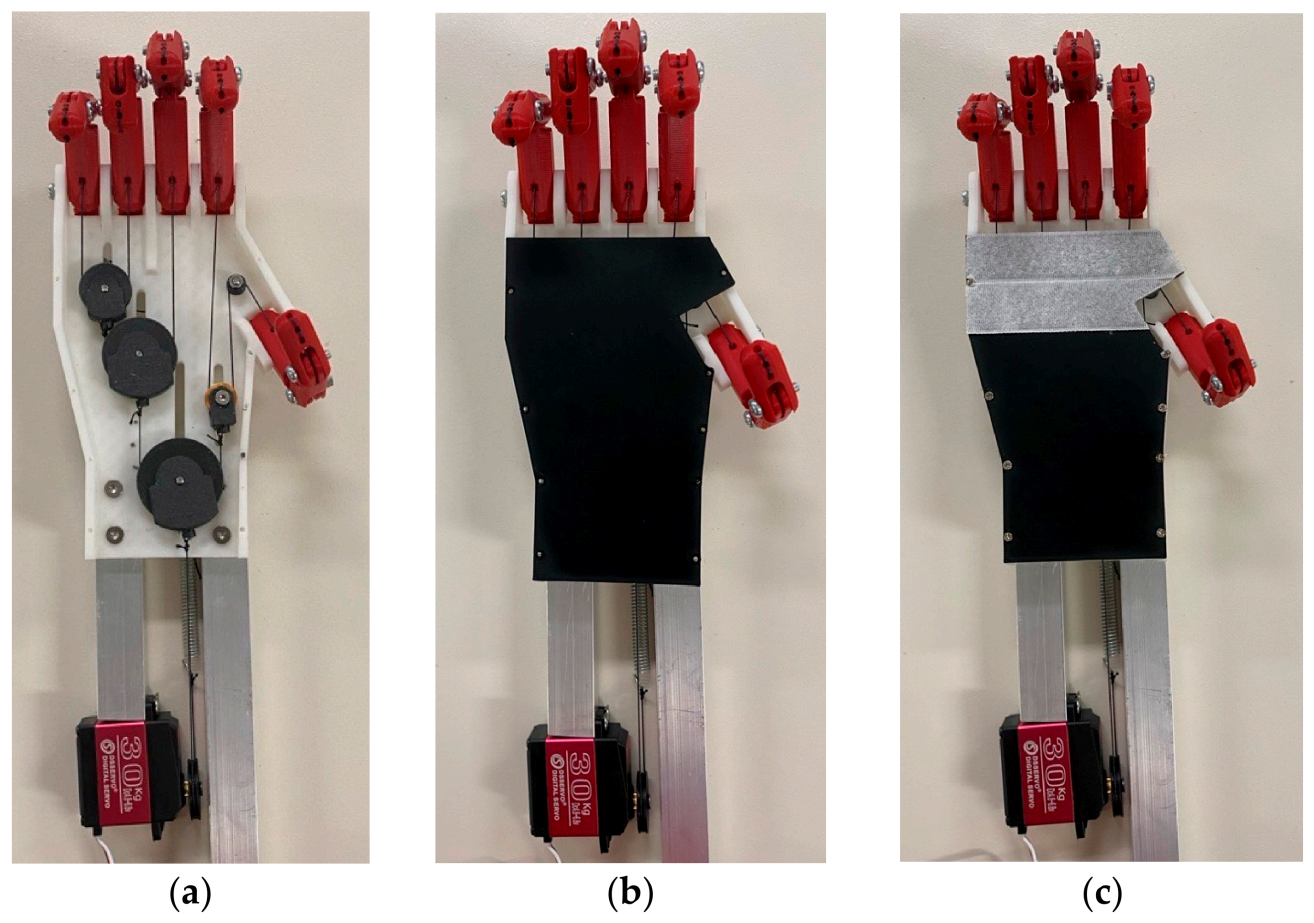
(**a**) The assembled Federica hand with the servo motor attached; (**b**) the Federica hand with a printed cover to allow objects to be placed in the hand; (**c**) the Federica hand with Velcro added to stop the “handlebar” from twisting when testing.

Once assembled, the Federica hand is mounted to a rigid frame along with the servo motor used for actuation (Figure 7a). The motor used was a DsServo 30 kg Metal Gear Digital Servo, Dongguan City Dsservo Technology Co., Ltd., Dongguan, China, chosen to match the torque rating of the original [14], which is attached to 100 mm × 19.5 mm^2^ square extruded aluminium. Once the motor is mounted, a 3D-printed cover is screwed onto the hand (Figure 7b) so items can be placed on it without interfering with the actuation mechanism. As per the 2021 paper in which the grip force of the Federica hand was analysed, a 1 kg KF100 load cell was placed inside what was referred to as the “handlebar” [14]. To measure the force exerted on the handlebar without variance due to torsion, Velcro was added to the handlebar and the Federica hand (Figure 7c). Details of the pulley installation are not covered here, as they were covered in previous work discussed in the introduction.

A linear regression method was used to calibrate the load cell, as was done in the original paper [14]. Four known masses were used, and the static measurements are shown in Table 5. This yielded a sensitivity of −530.53 µV/g, with the negative value being due to the fact that the voltage decreased as the load increased.

### 2.5. “Handlebar” Grip Force Test Setup

**Figure 8:**
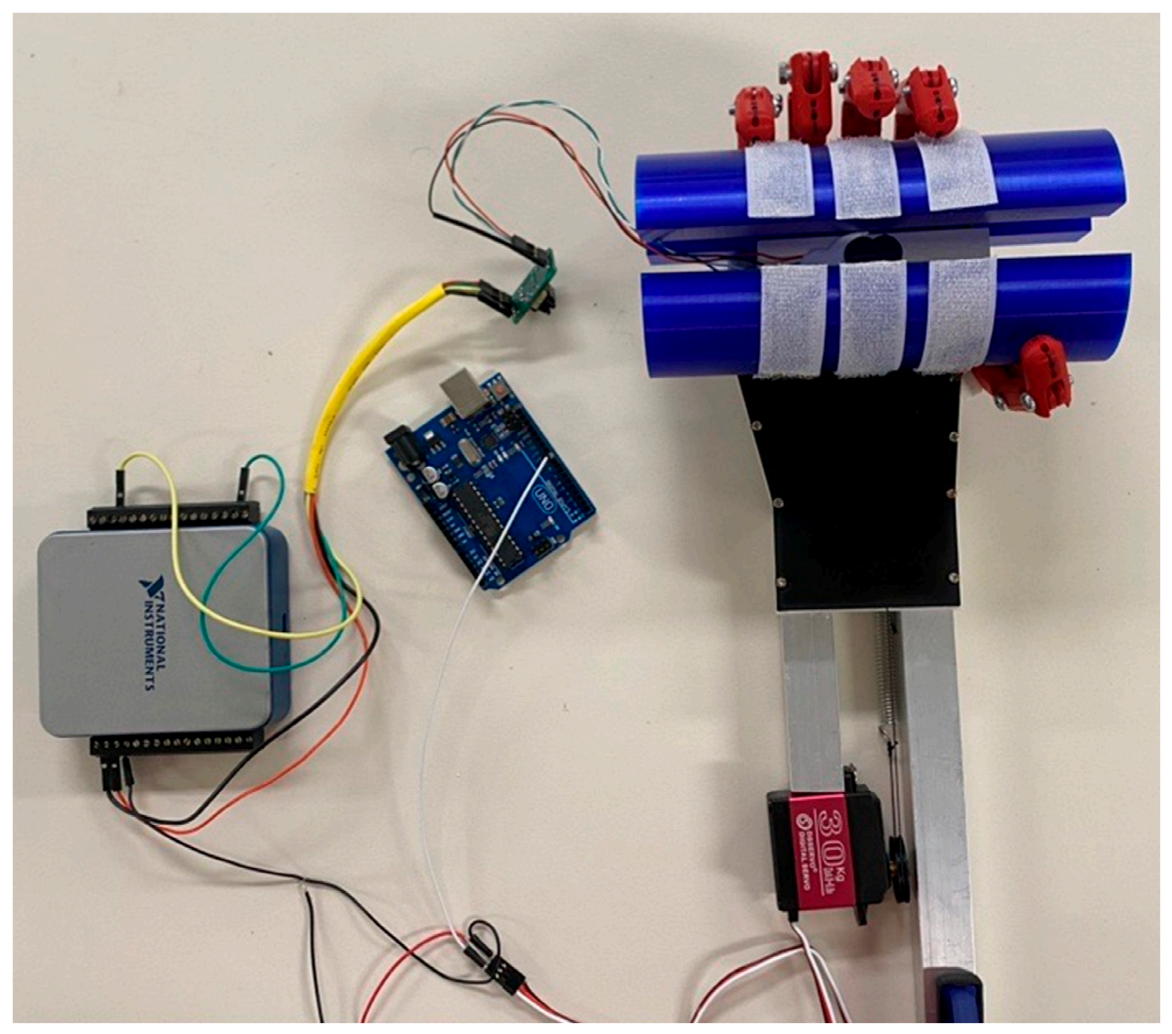
The test setup for the Federica hand includes the handlebar connected to the INA126 and the USB6002, and the Arduino Uno connected to the servo motor.

The grip-force measurement system used in the original Federica hand design was also implemented for the PIP Federica hand (Figure 8). The KF100 load cell was calibrated using a known weight. The load cell was connected to an instrumentation amplifier (INA126) to amplify the signal. The INA126 had a 1 kΩ gain resistor, which produced a gain of 85 V/V. This signal was then measured using a National Instruments USB-6002 DAQ, National Instruments, Austin, TX USA, which also provided a 2.5 V reference voltage for the INA126, Texas Instruments, Dallas, TX, USA. USB-6002 DAQ used a sampling rate of 100 Sa/s and a 16-bit resolution. The servo motor’s position was controlled using an Arduino Uno, Arduino, Monza, Italy.

**Figure 9:**
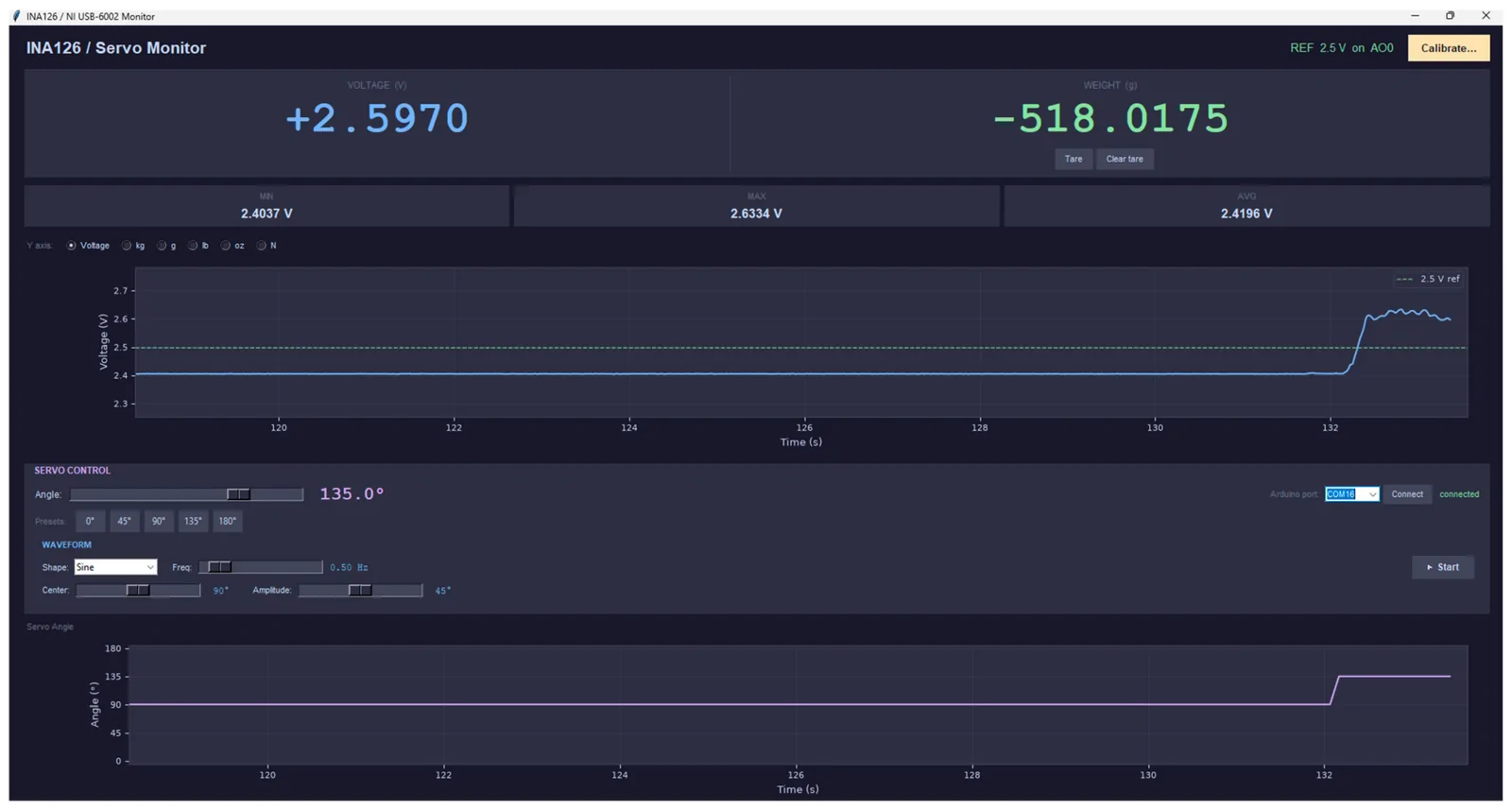
A screenshot of the interface of the Python code used to measure the force on the load cell and control the angle of the servo motor.

Measurement of motor force and control was managed using Python code, version 3.14 (Figure 9), and data was recorded and exported to an Excel spreadsheet for processing. To maintain consistency across tests, the servo motor was controlled via a square wave alternating between 45° and 180°. Only one PIP Federica hand and one original Federica hand were fabricated and tested in eight different orientations, making this a single-specimen analysis. This shows that the PIP hand performs comparably to the original Federica design.

**Figure 10:**
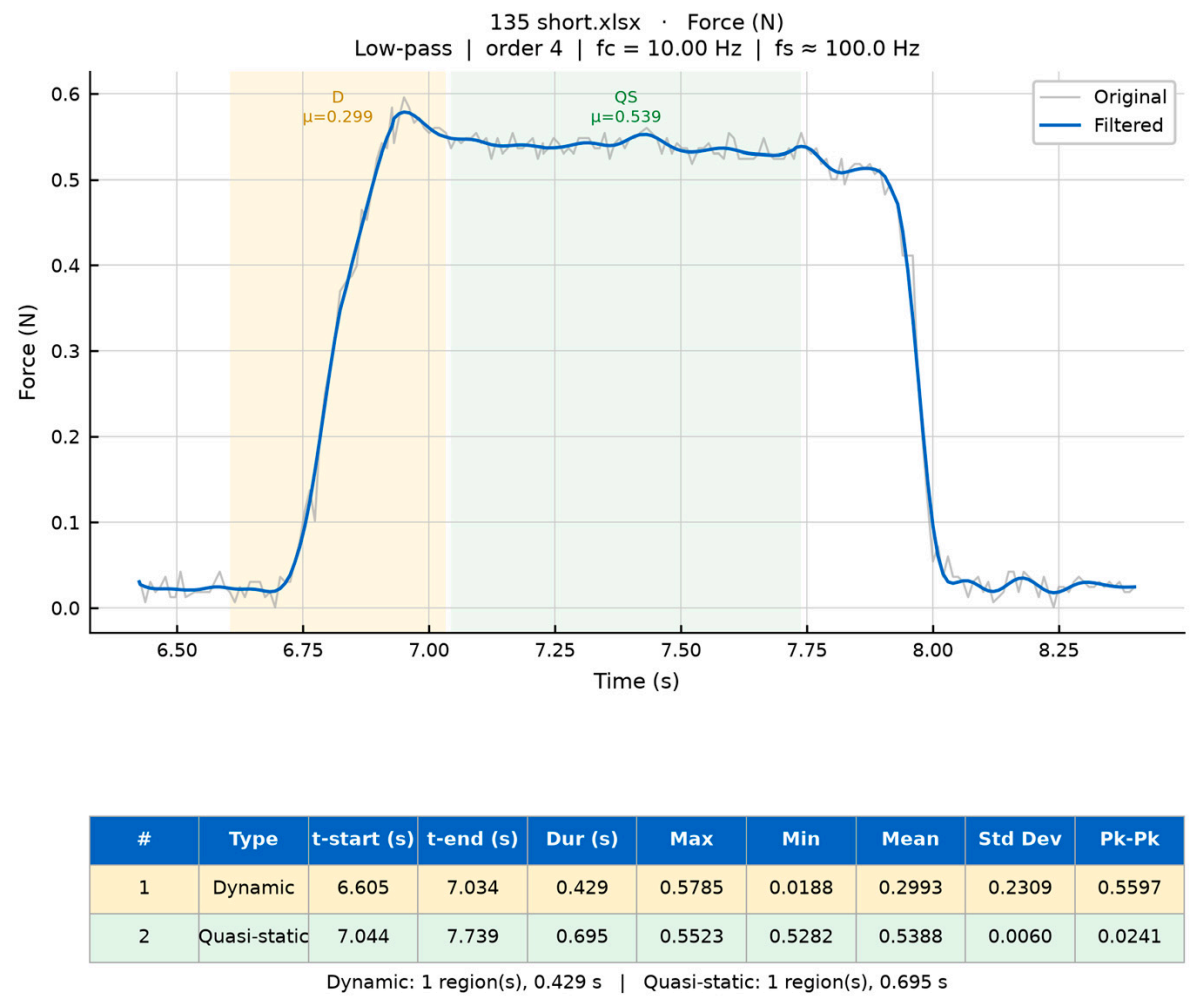
Depiction of the dynamic and static regions of the applied force after being filtered through the Butterworth filter.

After capturing the data, the file was run through another Python Code for processing (Figure 10). The code applied a 4th-order Butterworth low-pass filter with a 10 Hz cutoff to remove high-frequency noise from the signal. An offset was also added to remove the DC component of the signal, and graphs were synchronised manually. The code also divided the signal into dynamic and quasi-static regions, so metrics can be taken as the hand closes and while the hand holds its position.

**Figure 11:**
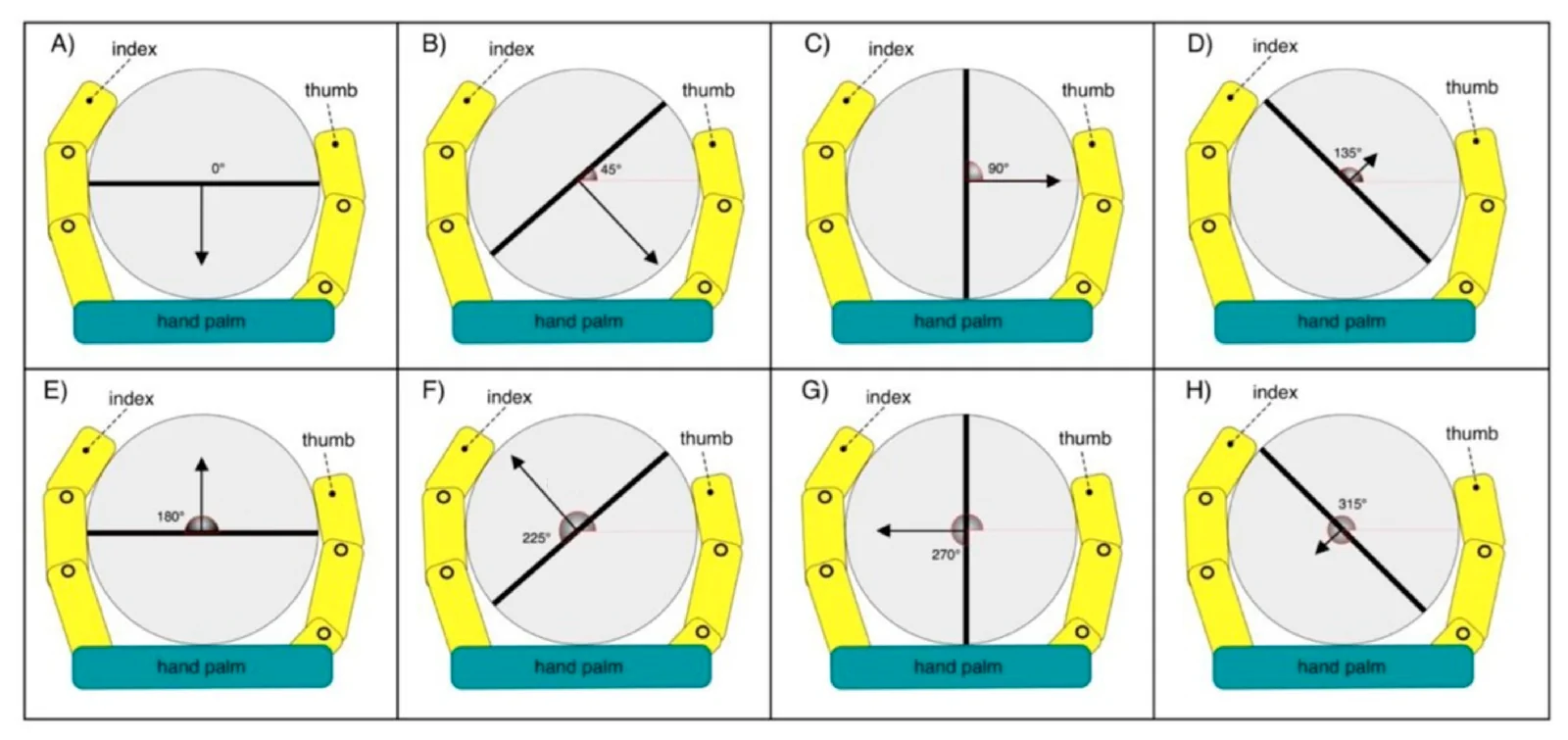
A depiction of the handlebars’ angle of rotation in the Federica hand, where the arrow indicates the angle at which the force is being measured, adapted from [14], at (**A**) 0°, (**B**) 45°, (**C**) 90°, (**D**) 135°, (**E**) 180°, (**F**) 225°, (**G**) 270°, and (**H**) 315°.

Since the load cell measures compression force along only one axis, measurements were taken at 8 different rotational positions (Figure 11), as per the original paper [14]. Force data was captured at 0°, 45°, 90°, 135°, 180°, 225°, 270°, and 315°.

## 3. Results

### Force Analysis

**Figure 12:**
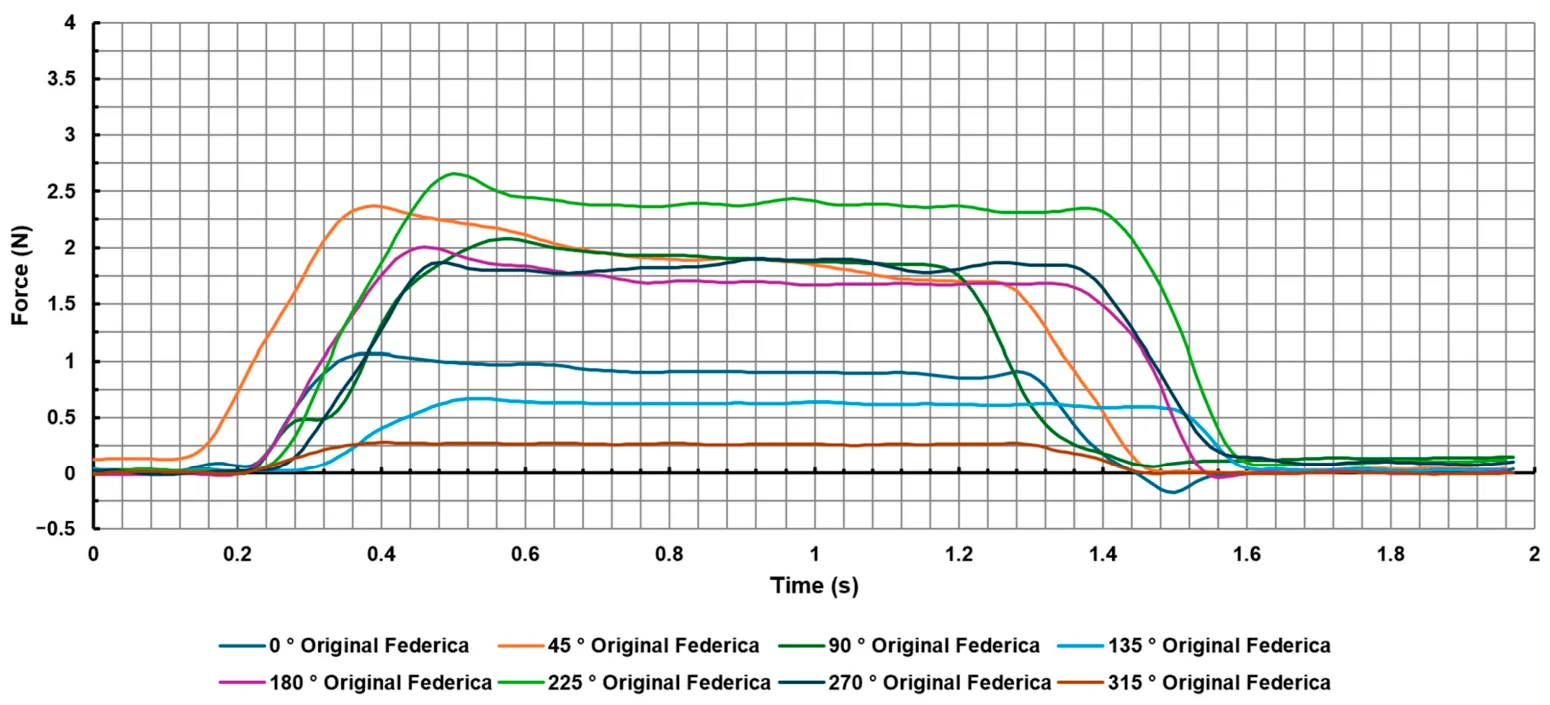
Grip force recorded with time for different angles of the load cell for the Original Federica hand.

In order to gauge the magnitude of the difference in grip strength between the ‘original’ Federica hand and the PIP version, both versions were tested concurrently. The data collected from testing of the Original Federica hand with the load cell at each orientation are displayed in Figure 12 and Table 6. Overlaying the data from each angle, we see the highest gripping force recorded with the handlebar orientated at 270°, with a maximum force of 2.65 N with the dynamic movement of the hand and a static mean force of 2.38 N when in the static holding position. Whilst the recorded force differs between these results and the 2021 results, this is consistent with the findings in the original test paper [14].

**Figure 13:**
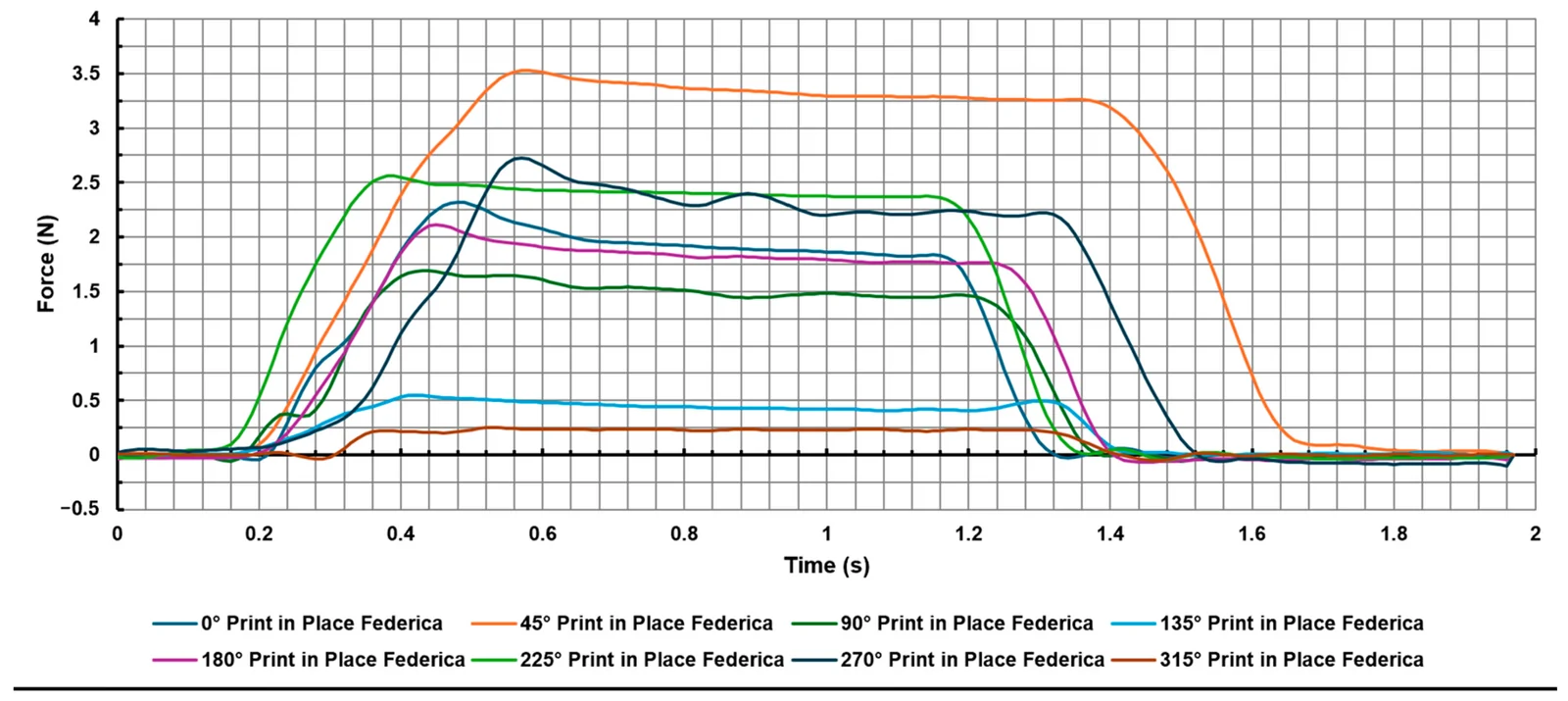
Grip force recorded over time for different angles of the load cell for the PIP Federica hand.

Figure 13 shows the grip force recorded for the PIP hand, similarly overlaid, with Table 7 showing the key metrics in both the dynamic and quasi-static moments of the hand. Again, the highest force is recorded with the handlebar oriented at 45°, where the maximum force during the kinetic movement was 3.53 N, with a mean force of 3.33 N in the static holding of the handlebar.

## 4. Discussion

### 4.1. Analysis of Grip Force Measurement Results

As this is a single-specimen design comparison, the goal of this analysis is to demonstrate comparable results. Achieving this level of force with a 3D-printed, under-actuated prosthetic such as the Federica hand brings multiple complications; actuation strength notwithstanding, concerns such as material and joint strength must be considered, as well as a material’s friction coefficient. In addition, the grip forces as tested were universally measured significantly lower than those in the 2021 paper [14]. However, through concurrent testing of a reproduction of the original Federica hand alongside the redesigned PIP version, a reasonable approximation of the PIP version’s efficacy can be obtained. This could be influenced by many factors; one of the issues is related to the closing speed of different parts of the phalanges.

When the hand is closing, the distal and medial phalanges often close before the proximal phalange has closed. This means the ends of the phalanges often apply the force to the load rather than the whole phalange. This could potentially be the reason less force was applied as opposed to the 2021 results [14]. To resolve this, future work could add a flexible sleeve to the phalanges to dampen the movement of the distal and medial phalanges so they can move with the proximal phalange. To keep with the affordable 3D-printed design of the Federica hand, this could be achieved using TPU (Thermoplastic Polyurethane) of varying stiffness and thickness.

### 4.2. Comparison of the Original Federica Hand and the Print-in-Place Federica Hand

**Figure 14:**
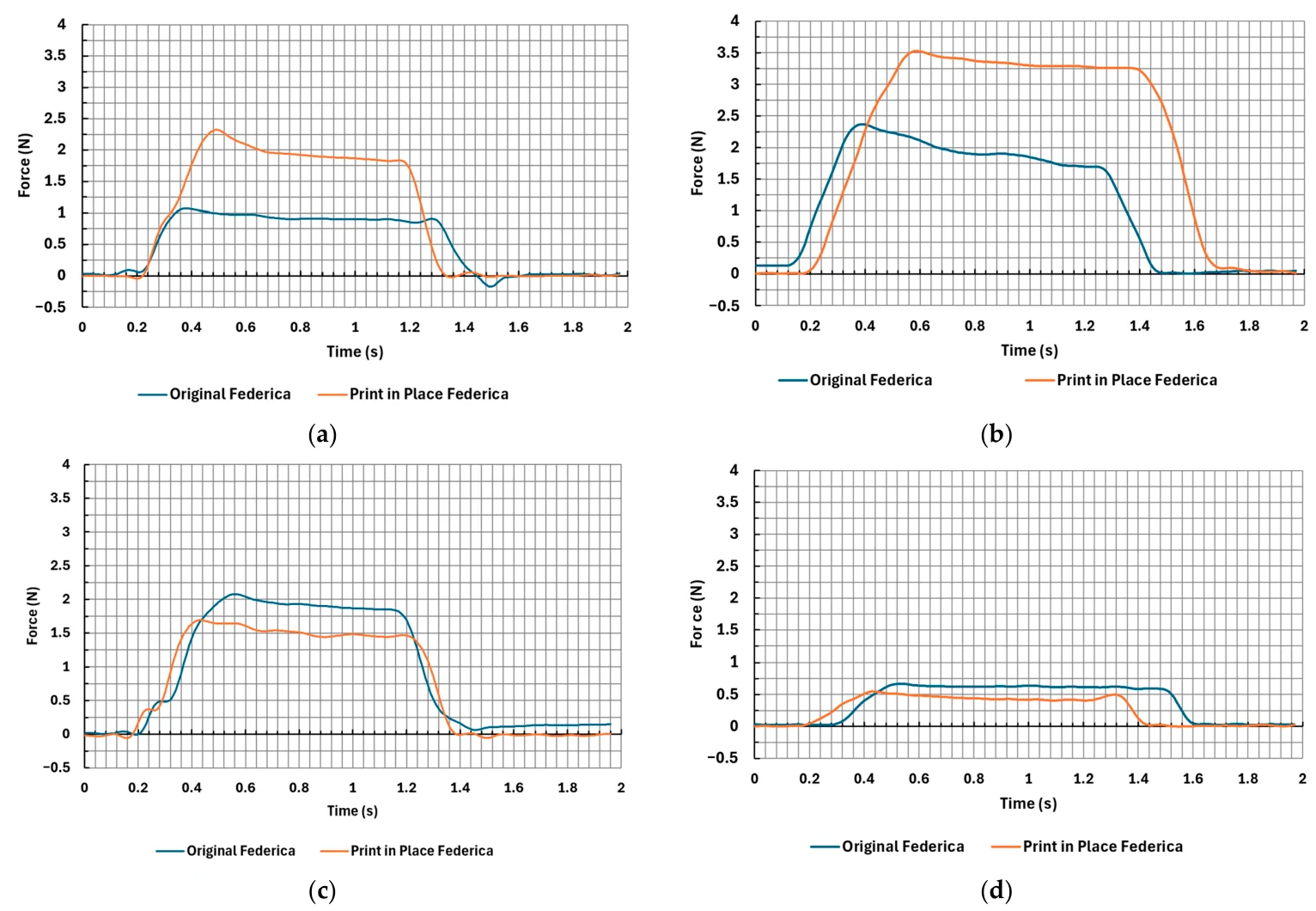

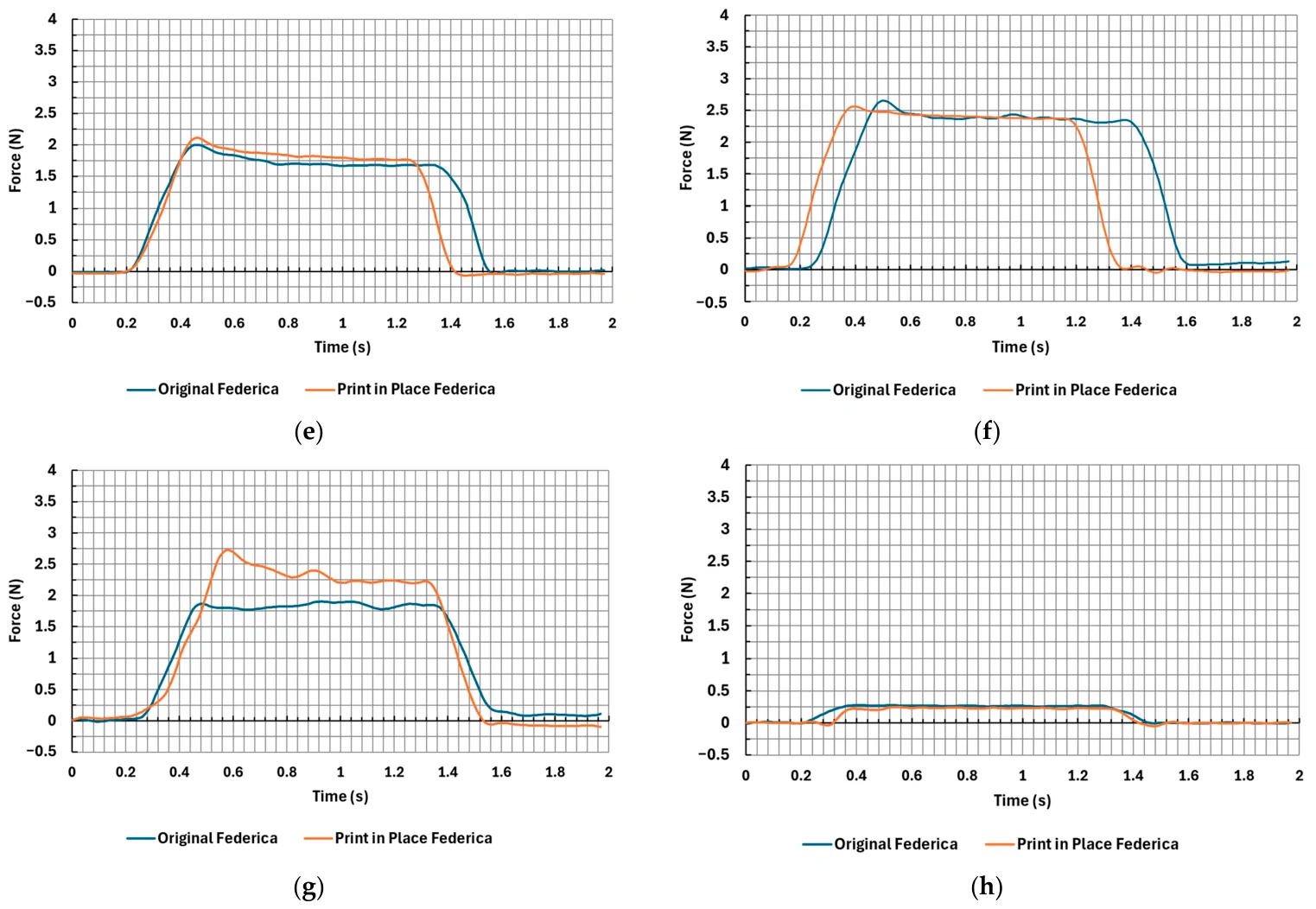
Comparison of the original Federica hand and the PIP Federica hand with the force applied at the following angles to the load cell: (**a**) 0°, (**b**) 45°, (**c**) 90°, (**d**) 135°, (**e**) 180°, (**f**) 225°, (**g**) 270°, and (**h**) 315°.

Figure 14a–h show the differences in grip force achieved between the ‘original’ and PIP hands, at each handlebar orientation. Observing the outputs side by side, we can see that the PIP Federica hand performs better than the original hand 50% of the time. Table 8 shows that the PIP produced dynamic forces from 70% of the original hand’s peak dynamic force up to 217% of that force. On average, the PIP produced 119% of the dynamic force produced by the original Federica. Table 8 also shows that the PIP Federica produced a static holding force between 60% and 212% of the original hand, and, on average, 18% higher than the original design. Even though the force was higher on average, it is not statistically significant enough to claim higher performance in a single-specimen analysis.

These differences in force between the specimens could be due to a few factors. The reduction in weight from the bolts increases the losses of the phalanges when a force is applied. The friction of the bolts on the phalanges will also add losses to the applied force of the phalanges. Though the forces demonstrated in this single-specimen design were notably below those shown in the 2021 paper [14], the absence of any such issues is encouraging regarding the PIP version’s feasibility. It should be noted that after the PIP Federica hand was run through 5000 cycles, there was no visual damage to the operation of the hand.

### 4.3. Future Work

Though the PIP Federica hand simplifies the Federica design, there are many areas for future research. To overcome the rigidity and mechanical strains of the PLA, new materials should be tested and compared, including PETG (polyethene terephthalate glycol) and reinforced carbon fibre material to improve the strength of the joints and to minimise the risk of joint failure during operation. Also, as discussed above, implementing mechanical dampers on the proximal phalanges to prevent pulling in too early would be a good addition. This could be done in TPU to keep the whole project PIP 3D-printed. Finally, even with the assembly time reduction in the PIP, the threading of the pulley is still quite labour intensive. Finding a practical way to reduce this aspect of assembly is important for wider adoption of the Federica hand.

## 5. Conclusions

The design and development of the print-in-place Federica hand addresses many of the constraints associated with traditional prosthetic assembly. By adding the print-in-place joints with a new locking mechanism, along with the addition of custom support material to the hand, the Federica hand could be successfully printed in a single assembly, directly on the print bed. The complete removal of the screws in the finger joints does not hinder the hand’s functionality. This highlights the balance that needs to be struck between simplicity, functionality, and user-centred innovation in the development of prosthetic devices; this requires iterative design and testing. Results from these efforts give a solid foundation for further development of open-source 3D-printed prosthetic hands.

## Figures and Tables

**Table 1:** Comparison of fabrication processes for the PIP Federica hand and original Federica hand. Both prints were configured in PLA, with a 0.4 mm nozzle, 0.2 mm layer height and a 15% infill.

	PIP Federica Hand	Original Federica Hand
Print time (h:min)	5:00	4:02
Hand components	1	16
Pulley and levels	16	16
Number of fasteners	5	17
Print supports	20	0
Filament required (g)	123.76	110
Pulley and lever assembly time (min:s)		

**Table 2:** Comparison of PIP Federica hand and original Federica hand samples, including weight and assembly time. This does not include the time required to attach and thread the pulleys, as that process is common to both designs. The weight of the pulleys and levers was also not included.

	PIP Federica Hand		Original Federica Hand	
	Support Removal Time (min:s)	Weight (g)	Assembly Time (min:s)	Weight (g)
1	1:09	84.75	10:26	109.25
2	1:07	86.48	13:36	109.16
3	1:15	86.71	12:43	107.96
Average	1:10	85.98	12:15	108.79

**Table 3:** Bill of materials for the items in the original Federica hand and their required quantities and costs. Two totals were provided: one for if all items needed to be purchased for an initial fabrication and the other for the unit cost assuming you have stock on hand.

Item	Quantity Required	Packet Size	Packet Cost (AUD)	Unit Cost (AUD)	Total Cost (AUD)
Printed hand structure (Bambu Lab PLA) [18]	110 g	1 kg	45.95	0.046	5.05
M4 × 15 mm bolt (finger joints) [19]	10	18	4.12	0.229	2.29
M4 × 20 mm bolt (thumb to palm) [20]	1	16	3.98	0.249	0.25
M4 × 80 mm bolt (knuckle through-rod) [21]	1	12	3.23	0.269	0.27
M3 × 20 mm bolt (threads into pulley) [22]	5	12	4.33	0.361	1.80
Nylon braided thread, 1 mm (tendon) [23]	1	1	0.80	0.800	0.80
		First Purchase Cost (AUD)	62.41	Total Cost (AUD)	10.47

**Table 4:** Bill of materials for the items in the PIP Federica hand and their required quantities and costs.

Item	Quantity Required	Packet Size	Packet Cost (AUD)	Unit Cost (AUD)	Total Cost (AUD)
Printed hand structure (Bambu Lab PLA) [18]	124 g	1 kg	45.95	0.046	5.69
M3 × 20 mm bolt (threads into pulley) [22]	5	12	4.33	0.361	1.80
Nylon braided thread, 1 mm (tendon) [23]	1	1	0.80	0.800	0.80
		First Purchase Cost (AUD)	51.08	Total Cost (AUD)	8.29

**Table 5:** Calibration results measured across four known masses, including 0 g, 50 g, 100 g, 150 g, and 200 g.

Known Mass (g)	Measured Voltage (V)
0	2.2366
50	2.2101
100	2.1836
150	2.1570
200	2.1305

**Table 6:** Key metrics from the data captured for the Original Federica hand, with both dynamic and quasi-static movement.

	Dynamic		Quasi-Static	
Rotation	Max (N)	Peak to Peak (N)	Max (N)	Mean (N)
**0**	1.07	1.06	0.99	0.92
**45.00**	2.37	2.24	2.25	1.92
**90.00**	2.08	2.09	2.06	1.92
**135.00**	0.67	0.65	0.64	0.63
**180.00**	2.00	2.03	1.87	1.71
**225.00**	2.65	2.64	2.48	2.38
**270.00**	1.86	1.85	1.89	1.83
**315.00**	0.28	0.29	0.27	0.27

**Table 7:** The key metrics from the data captured for the PIP Federica hand, with both Dynamic and Quasi-static movement.

	Dynamic		Quasi-Static	
Rotation	Max (N)	Peak to Peak (N)	Max (N)	Mean (N)
**0**	2.32	2.37	2.21	1.95
**45.00**	3.53	3.52	3.50	3.33
**90.00**	1.69	1.74	1.65	1.51
**135.00**	0.47	0.47	0.44	0.38
**180.00**	2.11	2.14	1.95	1.83
**225.00**	2.56	2.57	2.48	2.41
**270.00**	2.71	2.58	2.49	2.30
**315.00**	0.25	0.28	0.24	0.23

**Table 8:** Side-by-side comparison of PIP and original Federica hands demonstrating their maximum output in the dynamic region and mean force in their quasi-static regions.

	Dynamic			Quasi-Static		
Rotation	Original Federica Max (N)	PIP Federica Max (N)	PIP/Original Force Ratio (%)	Original Federica Mean (N)	PIP Federica Mean (N)	PIP/Original Force Ratio (%)
**0**	1.07	2.32	217%	0.92	1.95	212%
**45.00**	2.37	3.53	149%	1.92	3.33	173%
**90.00**	2.08	1.69	81%	1.92	1.51	79%
**135.00**	0.67	0.47	70%	0.63	0.38	60%
**180.00**	2.00	2.11	106%	1.71	1.83	107%
**225.00**	2.65	2.56	97%	2.38	2.41	101%
**270.00**	1.86	2.71	146%	1.83	2.30	126%
**315.00**	0.28	0.25	89%	0.27	0.23	85%
**Average**	1.62	1.96	119%	1.45	1.74	118%

## Data Availability

The data presented in this study are available in the above tables and figures, as well as the FigShare repository in the above Supplementary Materials.

## Supplementary Materials

All the information required to reproduce the “Federica” hand, including the print-in-place version, is available at: https://doi.org/10.6084/m9.figshare.13480248 (accessed on 25 August 2026).
